# Rupture du tendon rotulien avec fracture fermée distale du fémur homolatéral

**DOI:** 10.11604/pamj.2019.32.149.17723

**Published:** 2019-03-26

**Authors:** Soufiane Aharram, Yahyaoui Mounir, Abdelhafid Derfoufi, Abdessamad Kharraji, Jawad Amghar, Mohammed Benhamou, Lamhaoui Abdessamad, Bouziane Walid, Sadougui Mohammed, Omar Agoumi, Abdelkarim Daoudi

**Affiliations:** 1 Service de Traumatologie-orthopédie, CHU Mohammed VI, Faculté de Médecine et de Pharmacie d'Oujda, Oujda, Maroc

**Keywords:** Fémur distal, clou rétrograde, rupture du tendon rotulien, cerclage, Distal femur, retrograde femoral nail, patellar tendon rupture, cerclage wires, Fémur distal, clou rétrograde, rupture du tendon rotulien, cerclage

## Abstract

Un patient de 45 ans a subi un traumatisme du genou droit à la suite d'un accident de la voie publique occasionnant chez lui une fracture distale supra condylienne comminutive du fémur droit et une rupture du tendon rotulien ipsilatérale. Cette association est exceptionnelle et aucun cas n'a été retrouvé dans la littérature. Un diagnostic précis clinique et radiologique suivi d'une prise en charge précoce et adaptée par une ostéosynthèse interne et une rééducation fonctionnelle précoce et adaptée ont permis d'obtenir de bons résultats à long terme.

## Abstract (en)

We here report the case of a 45-year old patient who had injured his right knee because of a road accident. The patient had right comminuted supracondylar femur fracture and ipsilateral patellar tendon rupture. This association is exceptional and no case has been reported in the literature. Targeted clinical and radiological diagnostic tests followed by early and suitable management based on internal osteosynthesis and suitable early functional rehabilitation enabled good long term outcome.

## Introduction

Les fractures du fémur et du tibia provoquent une blessure au genou flottant. Fréquemment associées à d'autres lésions traumatiques, elles peuvent passer initialement inaperçues. Ils sont presque toujours dus à des traumatismes à haute énergie. Par conséquent, ils sont fréquemment associés à d'autres lésions concomitantes du membre ipsilatéral ou d'autres parties du corps, dont les lésions des ligaments du genou ipsilatéral sont importantes pour diverses raisons. Nous rapportons le cas d'une fracture fermée comminutive supra condylienne du fémur droit associée à une rupture complète du tendon rotulien ipsilatérale.

## Patient et observation

**Figure 1:**
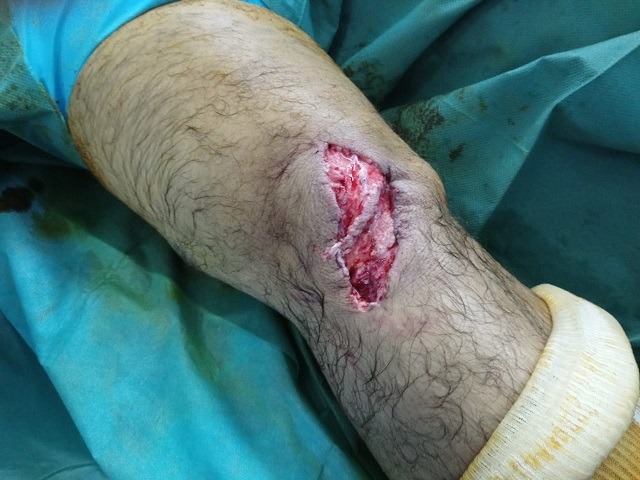
aspect clinique à l’admission du patient au bloc opératoire

**Figure 2:**
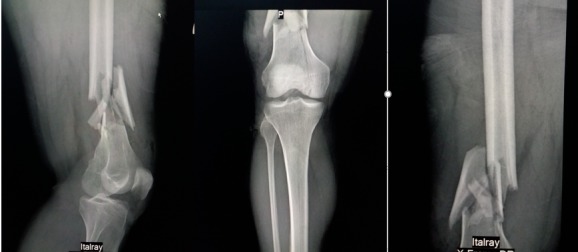
radiographie du genou face montrant une fracture supra condylienne avec un trait de refend sur le condyle médial, cependant sur le profil on note une ascension de la rotule

Il s'agit d'un patient âgé de 45 ans, sans antécédents pathologiques particuliers, qui a été admis aux urgences à la suite d'un accident de la voie publique (reversement d'une voiture) occasionnant un traumatisme par choc direct sur le genou droit. Le patient avait présenté une plaie oblique profonde de 5cm sans perte de substance cutanée localisé à la face antérieure du genou droit associée à un gonflement et une impotence fonctionnelle totale du membre inferieur droit. L'examen clinique a mis en évidence une plaie propre en regard du tendon rotulien associé à une douleur et un gonflement du genou droit, une sensibilité et une déformation de la cuisse distale, l'examen vasculo-nerveux du membre inférieur droit était normal et le reste de l'examen physique du patient était sans particularité (Figure 1). Des radiographies du genou droit de face et de profil ont été réalisées en urgence et ont mis en évidence une fracture supra condylienne du fémur droit comminutive avec un trait de refend sur le condyle médial et une bonne congruence articulaire sur la face (Figure 2).

**Figure 3:**
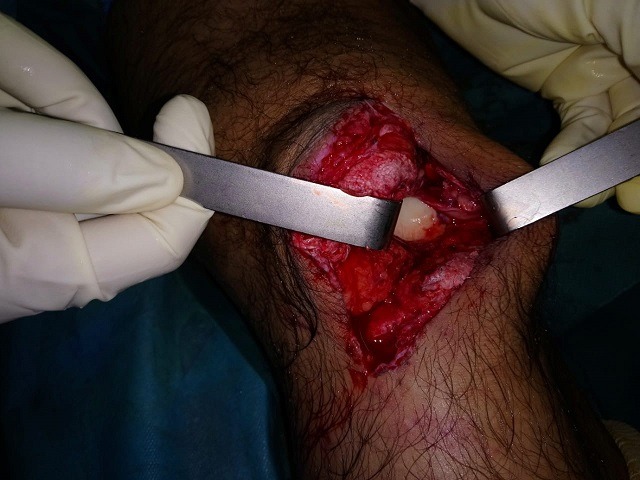
aspect clinique de la rupture transtendineuse en peropératoire après parage de la plaie

Le patient a bénéficié d'un acte chirurgical à H2 de son admission sous rachianesthésie d'un parage lavage de la plaie avec excision des tissus douteux. Le bilan lésionnel a objectivé une rupture complète du tendon rotulien, section de la capsule articulaire, un petit arrachement osseux de 0,5cm du plateau tibial externe, un trait de refend au niveau du condyle interne sans déplacement, les ligaments croisés ont été intactes (Figure 3).

Nous avons profité de l'ouverture de la capsule articulaire pour repérer le point d'introduction qui correspond au sommet de l'échancrure inter condylienne puis mise en place d'un clou rétrograde avec double verrouillage (proximal et distal). Le tendon rotulien a été libéré sur toute sa longueur et toutes ses faces. Les extrémités du tendon rompu ont été rapprochées et suturées par un laçage selon la technique de Judet [1]. Pour protéger la suture, un cadrage du tendon rotulien avec un gros fil d'acier métallique passant dans la rotule et la tubérosité tibiale antérieur (TTA) a été mis en place.

**Figure 4:**
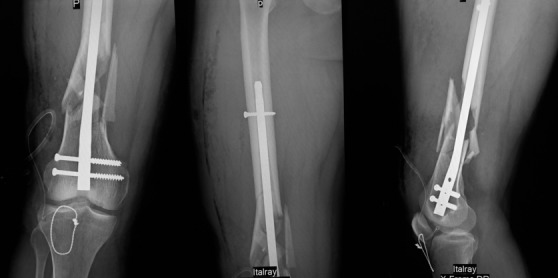
radiographies de contrôle en post opératoire de face et de profil à 30° de flexion avec un indice de Caton et Descamps à 1,1

**Figure 5:**
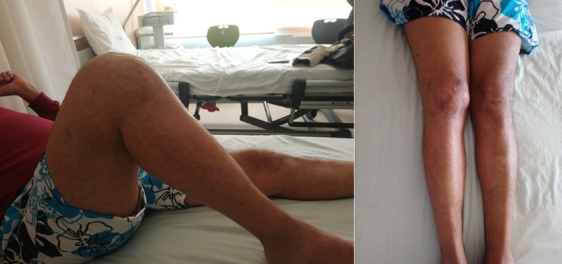
les amplitudes articulaires du genou après la 8^ème^ semaine de l´intervention

Un contrôle radiographique de profil à 30° de flexion comme recommandée par Ait Si Selmi [2] a été réalisée pour régler la hauteur rotulienne selon l'indice de Caton et Descamps (Figure 4). Une immobilisation par attelle amovible a été utilisée pour une durée de 45 jours, avec un traitement médical adapté. La marche sans appui a été autorisée dès le lendemain avec l'utilisation de béquilles. La rééducation fonctionnelle a débuté précocement au 3^ème^ jour post opératoire en favorisant la flexion passive, limitée à 70° pendant 45 jours. Le patient a été revu régulièrement avec contrôle clinique et radiographique. Après un recul de 8 semaines nous avons évalué l'évolution de la suture selon les critères de Siwek et Rao JP [3] se basant sur l'étude de deux éléments: l'amplitude articulaire et la force du quadriceps. Selon ces critères l'évolution clinique de notre patient a été jugée bonne (Figure 5).

## Discussion

Les fractures distales du fémur sont caractérisées par une grande variabilité anatomique, une difficulté d'évaluation radiologique et une approche thérapeutique controversée [4, 5]. Elles surviennent après un impact direct, une avulsion ou une force de cisaillement sur le genou, généralement secondaires à des accidents sportifs ou de la voie publique, notamment un traumatisme de tableau de bord [6, 7]. Par conséquent, il est généralement associé à de multiples blessures au même membre ou à d'autres parties du corps. Parmi les blessures associées aux traumatismes du genou, l'implication des ligaments du genou ipsilatéraux est particulièrement importante car ils passent le plus souvent inaperçus et ont contribué à des résultats plus médiocres dans la plupart des études. Toutes les blessures ligamenteuses ont été détectées et réparées au moment même de la fixation de la fracture [8].

La lésion au ligament croisé antérieur (LCA) était la découverte la plus fréquente. Et ils ont préconisé une évaluation minutieuse du genou dans tous les cas de fracture du fémur et en particulier lorsque le tibia est également fracturé. L'examen physique révèle généralement un œdème, un épanchement, ou des lésions cutanées (plaie, ecchymoses, éraflures) dans la région du genou, l'examen neurovasculaire du membre doit être soigneusement effectué. Le diagnostic radiographique sur des radiographies standards peut être difficile, et des incidences radiographiques antéro-postérieures, latérales et patellaires tangentielles, sont nécessaires pour confirmer le diagnostic et préciser les caractéristiques directs ou indirects de ces fractures.

En effet l'objectivation d'une rotule haute peut être rendu difficile par un aspect controlatéral semblable. De même la difficulté d'extension active de la jambe peut être masquée par des ailerons rotuliens intacts. Les examens radiologiques s'avèrent utiles pour la détection de patella alta. Dans notre cas le patient a présenté une plaie trans-tendon rotulien dont le diagnostic d'une rupture du tendon rotulien est obtenu cliniquement avec une fracture distale du fémur qu'était extra-articulaire.

Hung *et al.* ont également constaté que l'atteinte intra-articulaire du genou était le facteur le plus important contribuant à une issue défavorable [9]. Concernant le volet thérapeutique, deux difficultés sont à noter; la première réside dans l'absence de référence comparative pour la hauteur patellaire comme recommandé dans certaines publications. Il est essentiel de placer alors les deux rotules aux mêmes niveaux avec un indice de Caton [10] inférieur à 1,2 des ratios peropératoires, qui sont nécessaires pour assurer le réglage de la hauteur rotulienne.

Concernant l'attitude thérapeutique, le traitement chirurgical est le plus recommandé pour une fracture distale du fémur, il consiste en une réduction à foyer (fermé ou ouvert) suivie d'une stabilisation par différentes techniques, telles que des plaques, des vis condyliennes spongieuses et des vis canulées ou vis-plaque. Dans notre cas l'ostéosynthèse de la fracture supra-condylienne a été réalisée par un clou rétrograde avec double verrouillage (deux vis canulées 6,5 en distal et une vis corticale 4,5 en proximal). Plusieurs techniques ont été décrites dont le but est d'obtenir une réparation solide permettant de débuter rapidement la rééducation. Cette réparation repose sur une suture tendineuse directe ou une réinsertion transosseuse, protégée par un cadrage tendineux par un gros fil non résorbable (ou une allogreffe, notamment de type DIDT, en cas de perte de substance) [11-13]. La technique de laçage décrite par judet couplée à un cadrage provisoire protégeant la suture nous a donné des résultats satisfaisants.

## Conclusion

Nous rapportons le cas d'une lésion rare dans la littérature: rupture du tendon rotulien avec fracture fermée distal du fémur homolatéral pour souligner l'importance d'une enquête secondaire approfondie sur les lésions ligamentaires du genou, qui est souvent omise. Le mécanisme est généralement un traumatisme à haute énergie. Ces fractures doivent être traitées chirurgicalement avec une réduction anatomique et une ostéosynthèse interne stable suivie d'une rééducation fonctionnelle précoce afin d'obtenir de bons résultats fonctionnels à long terme.

## Conflits d’intérêts

Les auteurs ne déclarent aucun conflit d'intérêts.

## References

[1] Caldas MTL, Gustavo HSB, Manuela BFB (2013). Simultaneous bilateral rupture of the patellar ligament in chronicrenal patient, case report. Rev bras ortop.

[2] Ait Si Selmi T, Neyret P, Rongieras F, Caton J (1999). Ruptures de l'appareil extenseur du genou et fractures de rotule. techniques Chirurgicales-orthopédie-traumatologie.

[3] Siwek CW, Rao JP (1981). Ruptures of the extensor mechanism of the knee joint. The Journal of Bone and Joint Surgery.

[4] Smith EJ, Crichlow TP, Roberts PS (1989). Monocondylar fractures of the femur: a review of 13 patients. Injury.

[5] Nordin JY, Masquelet AC, Gavard R, Signoret F (1985). Les fractures unicondyliennes du fémur. Réflexions à partir d'une série de 90 observations. Rev Chir Orthop.

[6] Kennedy JC, Grainger RW, McGraw RW (1966). Osteochondral fractures of the femoral condyles. J Bone Joint Surg.

[7] Strauss E, Nelson JM, Abdelwahab IF (1984). Fracture of the lateral femoral condyle: a case report. Bull Hosp Joint Dis.

[8] Dickob M, Mommsen U (1992). Extra-articular fractures near the knee joint and concomitant damage. Aktuelle Traumatologie.

[9] Hung SH, Lu YM, Huang H, Lin YK, Chang JK, Chen JC (2007). Surgical treatment of type II floating knee: comparisons of the results of type IIA and type IIB floating knee. Knee Surgery, Sports Traumatology, Arthroscopy.

[10] Caton J, Deschamps G, Chambat P, Lerat JL, Dejour H (1981). Patella infera: apropos of 128 cases. Revue de chirurgie orthopedique et réparatrice de l'appareil moteur.

[11] Kim JR, Park H, Roh SG, Shin SJ (2010). Concurrent bilateral patellar tendon rupture in a preadolescent athlete: a case report and review of the literature. J Pediatr Orthop B.

[12] Van der Bracht H, Verdonk R, Stuyts B (2009). Augmentation of a patellar tendon repair with an autologous semitendinosus graft. Acta Orthop Belg.

[13] Milankov MZ, Miljkovic N, Stankovic M (2007). Reconstruction of chronic patellar tendon rupture with contralateral BTB autograft: a case report. Knee Surg Sports Traumatol Arthrosc.

